# Comparison of Laryngoscope-Guided Insertion and Standard Blind Insertion of the Laryngeal Mask Airway: A Systematic Review and Meta-Analysis

**DOI:** 10.1155/anrp/1224567

**Published:** 2025-03-18

**Authors:** Zhihao Zheng, Haibo Li, Congcong Dai, Liwei Bi, Wei Sun, Miao Yu, Zhanfei Hu, Xiaodong Liang

**Affiliations:** ^1^Department of Anesthesiology, Affiliated Chifeng Clinical Medical College of Inner Mongolia Medical University, Chifeng, Inner Mongolia, China; ^2^Department of Anesthesiology, Chifeng Municipal Hospital, Chifeng, Inner Mongolia, China

**Keywords:** airway management, anesthesia, blind insertion, laryngeal mask airway, laryngoscope guidance

## Abstract

**Background:** The insertion of a laryngeal mask airway (LMA) using a laryngoscope-guided technique has produced inconsistent outcomes. The use of laryngoscope guidance in facilitating LMA insertion needs further investigation. This meta-analysis compared its effectiveness and safety against standard blind insertion.

**Method:** We systematically searched PubMed, Cochrane Library, Web of Science, and Ovid Medline for randomized controlled trials comparing laryngoscope-guided (Group L) and blind LMA insertion (Group B). The primary outcome measured was fiberoptic staging. The secondary outcomes included oropharyngeal leak pressure (OLP), insertion time, success rate on the first attempt, and the incidence of postoperative sore throat and blood staining.

**Results:** Nine RCTs (1016 patients) were analyzed. The fiberoptic staging in Group L was found to be significantly higher than that in Group B (RR = 1.54; 95% CI: 1.14–2.08; *p*=0.005). In addition, the OLP of Group L is significantly higher than that of Group B (MD = 2.10 cmH_2_O; 95% CI: 0.38 cmH_2_O–3.83 cmH_2_O; *p*=0.02). The success rate for the first attempt was also higher in Group L (RR = 1.14; 95% CI: 1.06–1.22; *p*=0.0005). The insertion time (MD = 3.92 s; 95% CI: -6.69 s–14.52 s; *p*=0.47), the incidence of sore throat (RR = 0.90; 95% CI: 0.50–1.65; *p*=0.74), and the incidence of blood staining (RR = 1.19; 95% CI: 0.29–4.79; *p*=0.81) did not demonstrate statistically significant differences.

**Conclusion:** The use of LMA with laryngoscope guidance may improve fiberoptic staging and OLP and increase the success rate of first-attempt insertion, without significantly raising the incidence of sore throat or blood staining in anesthetized patients.

## 1. Introduction

The laryngeal mask airway (LMA) is extensively used in various clinical situations as a ventilation device [1, 2]. It is recognized as a safe and effective supraglottic airway device that may serve as an alternative to the endotracheal tube in certain adult and pediatric surgical procedures [3, 4]. The insertion technique for the LMA was initially described by Archie Brain, who recommended that the device be inserted until resistance was encountered [5]. Nevertheless, the standard blind insertion method may not allow the insertion of the LMA in the optimal position without complications. Common complications of unsuccessful LMA insertion include sore throat, hoarseness, and air leakage [6, 7].

Several studies have investigated the application of the laryngoscope to facilitate the insertion of LMA to avoid associated complications [8–11]. The results of LMA insertion using the laryngoscope-guided technique have yielded inconsistent findings. Kim GW et al.'s research revealed that the first attempt's success rate was comparable between standard blind and laryngoscope-guided insertion [11]. Similarly, Patil PC et al. demonstrated that laryngoscope-guided insertion is comparable to the blind insertion technique, as evaluated through fiberoptic assessment. The two methods did not show significant differences in hemodynamic stability and postoperative complications [12]. However, numerous studies examining different types of LMA have demonstrated that the efficacy of laryngoscope-guided insertion methods is superior to that of blind insertion techniques [8, 12, 13]. Furthermore, other studies have indicated that laryngoscope-guided techniques achieve higher success rates than blind methods [9, 14–16]. Consequently, the effectiveness of laryngoscope guidance in LMA insertion needs further investigation.

Fiberoptic staging is an effective method for assessing LMA insertion [17]. It has stages: Stage 1, inability to see vocal folds; Stage 2, vocal cords and anterior epiglottis visualized; Stage 3, vocal cords and posterior epiglottis visualized; and Stage 4, only vocal cords visible [18, 19]. The research conducted by Campbell et al. established five ordinal categories, designated as A–E, which correspond to the extent of the glottic aperture obscured by the epiglottis. Regarding the glottic fiberoptic view, Category A shows the epiglottis in a well-positioned and hidden state, while Categories B to E depicts the increasing glottic covering due to an improperly positioned epiglottis [9]. The measurement of oropharyngeal leak pressure (OLP) is critically important for assessing the safety and efficacy of the LMA [20, 21]. It is used to quantify the airtightness of various types of LMA, thereby indicating the level of airway protection [22–24]. In patients with obesity and those undergoing pneumoperitoneum, an elevated OLP is associated with improved efficacy of mechanical ventilation [24–29]. Together, fiberoptic staging and OLP provide crucial tools for clinicians to evaluate and ensure the proper functioning and suitability of the LMA in diverse clinical settings.

Due to the heterogeneity in sample sizes, research subjects, methodologies, and evaluation indicators across various studies, discrepancies and uncertainties have emerged in the research findings [30]. This meta-analysis aimed to synthesize data from multiple studies, provide a comprehensive summary of the evidence, enhance the support for conclusions, and compare the differences between laryngoscope-guided insertion and blind insertion of the LMA for fiberoptic staging, OLP, first insertion success rate, insertion time, and the incidence of postoperative complications.

## 2. Methods

We adhered to the Cochrane statistical method standards and the Preferred Reporting Items for Systematic Reviews and Meta-Analyses (PRISMA) statement [31]. On September 18, 2023, the protocol for this review was registered with the Prospective Register of Systematic Reviews (PROSPERO) network (registration number: CRD42023461675; https://www.crd.york.ac.uk/PROSPERO).

### 2.1. Data Sources and Search Strategy

The relevant studies were obtained from PubMed, Cochrane Library, Web of Science, and Ovid Medline databases, following the established methodologies. The search strategy integrated terms from EMTREE and Medical Subject Headings with free-text keywords. A detailed description of the search terms, which encompassed a combination of free text, Medical Subject Headings, and EMTREE terms, is provided in Appendix 1.

No restrictions were imposed regarding the year of publication. The most recent search was conducted on September 26, 2023. A manual search of the references cited in the selected articles was also performed. We also contacted the authors and experts in the field to obtain information about data that had yet to be published.

### 2.2. Selection of Studies

This meta-analysis considered controlled, quasirandomized, and randomized clinical trials (RCTs). The research selected adhered to the participants, population, intervention, comparator, and outcome (PICO) framework.. The inclusion criteria for the studies analyzed were as follows: (1) The anesthetist used the LMA to ventilate the patients during general anesthetic operations; (2) laryngoscope guidance as “intervention”; (3) The blind LMA insertion as “comparison”; (4) They reported at least one outcome indicator of the fiberoptic staging, OLP, the success rate of first insertion, insertion time, and the incidence of postoperative complications such as sore throat and blood staining. The exclusion criteria were as follows: (1) Studies involving children under 18 years of age and (2) studies that consisted of conference abstracts, unrelated research, editorials, duplicate publications, correspondence, editorial reviews, animal studies, models, off-topic studies, case reports, and retrospective studies.

The titles and abstracts of the studies identified through the previously mentioned search methods were meticulously reviewed by the three authors (HBL, LWB, and WS). The complete article was retrieved and analyzed if a study was deemed eligible based on its title or abstract. The same authors also discussed whether a study should be included or excluded from the review. All authors resolved disagreements regarding inclusion or exclusion through collaborative discussion.

### 2.3. Data Extraction

Three authors (CCD, ZFH, and MY) independently extracted the following data: the year of publication, the first author, the number of patients and their characteristics, the type of LMA used, and the type of laryngoscope employed. The primary outcome measured was fiberoptic staging. In addition, the first-attempt insertion success rate, OLP, the insertion time, and the postoperative complications, including postoperative sore throat and blood staining, were included as secondary outcomes.

### 2.4. Assessment of the Risk of Bias

Following a domain-based evaluation to assess the risk of bias, utilizing the Cochrane Collaboration Risk of Bias tool, three authors (HBL, ZFH, and XDL) assessed bias risks in the selected studies. This evaluation encompassed various factors, including partial outcome data, selective reporting, blinding personnel and participants, random sequence generation, allocation concealment, and other potential biases. Each domain was classified according to its risk of bias as low, unclear, or high. Any disagreements among the investigators regarding the eligibility of the studies, data extraction, and risk of bias assessment were resolved through consensus.

### 2.5. Evaluation of the Certainty of Evidence

To assess the quality of study sources, the modified Jadad scale was used (Table 1). Four criteria were utilized to evaluate the overall quality of the included studies: randomization (scoring from 0 to 2), blinding (scoring from 0 to 2), allocation concealment (scoring from 0 to 2), and withdrawals or dropouts (scoring from 0 to 1). A total score ranging from 4 to 7 indicated high quality, whereas a score between 1 and 3 suggested inferior quality. Higher scale scores, which range from 0 to 7, correspond to higher-quality assessments.

### 2.6. Data Synthesis and Analysis

Review Manager V.5.4 (RevMan, Cochrane Collaboration, Oxford, United Kingdom) was used for the analysis. The group difference was expressed as the random-effects Mantel–Haenszel risk ratio (RR) and a 95% confidence interval (CI) for dichotomous data. Differences between groups in continuous data were represented as mean difference (MD) with a 95% CI. The *I*^2^ statistic was used to assess the heterogeneity of the included studies. An *I*^2^ value of ≤ 50% and a *p* value of > 0.1 indicated homogeneity among the studies. The heterogeneity was significant if the *I*^2^ value was > 50% and the *p* value was < 0.1. A random-effects model was used. A significance level of *p* < 0.05 was considered statistically significant. Subgroup analysis was used to explore the sources of heterogeneity.

## 3. Results

### 3.1. Search Results

**Figure 1:**
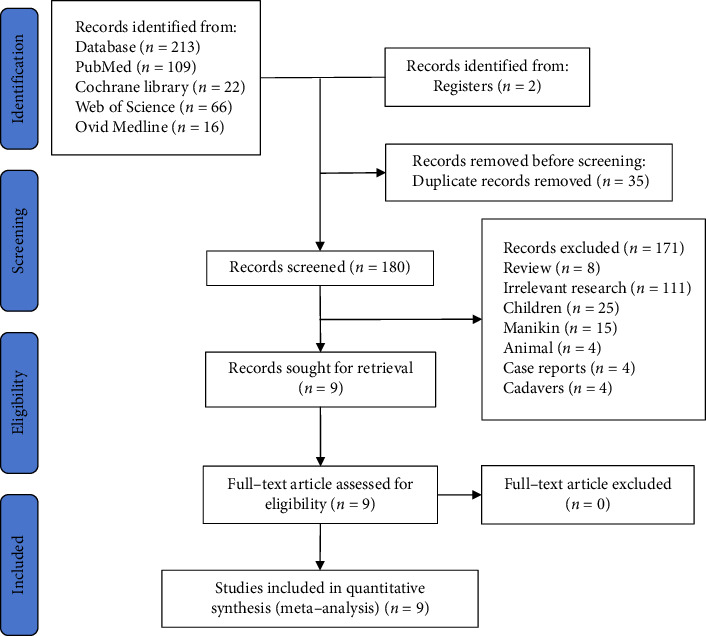
Flowchart of the trials included in the meta-analysis.

A total of nine studies involving 1016 patients were included in this meta-analysis [8–16]. Initially, 213 studies were evaluated through searches conducted in the PubMed, Cochrane Library, Web of Science, and Ovid Medline databases. Two studies were registered for inclusion. Following the removal of duplicates, 180 studies remained. After reviewing the titles and abstracts, 171 studies were excluded. Finally, a comprehensive review of the full texts of the remaining studies was conducted, and nine studies were included for analysis (Figure 1).

### 3.2. Characteristics of the Study

The characteristics of the nine studies are shown in Table 2. All studies were performed on patients classified as American Society of Anesthesiologists (ASA) Physical status I-II. Three types of laryngeal masks were evaluated: LMA–Flexible [11, 14], LMA–Classic [8–10, 13], and LMA–ProSeal [12, 15, 16]. In addition, two types of laryngoscopes were assessed: the Macintosh laryngoscope [8, 9, 11–15] and the video laryngoscope [11, 15]. The surgical procedures included dental surgery, elective orthopedic or general surgical procedures, ambulatory surgery, oral, maxillofacial, ENT, and general surgery. Muscle relaxants were used in two studies [12, 15] during the insertion of the LMA.

### 3.3. Assessment of Bias Risk

**Figure 2:**
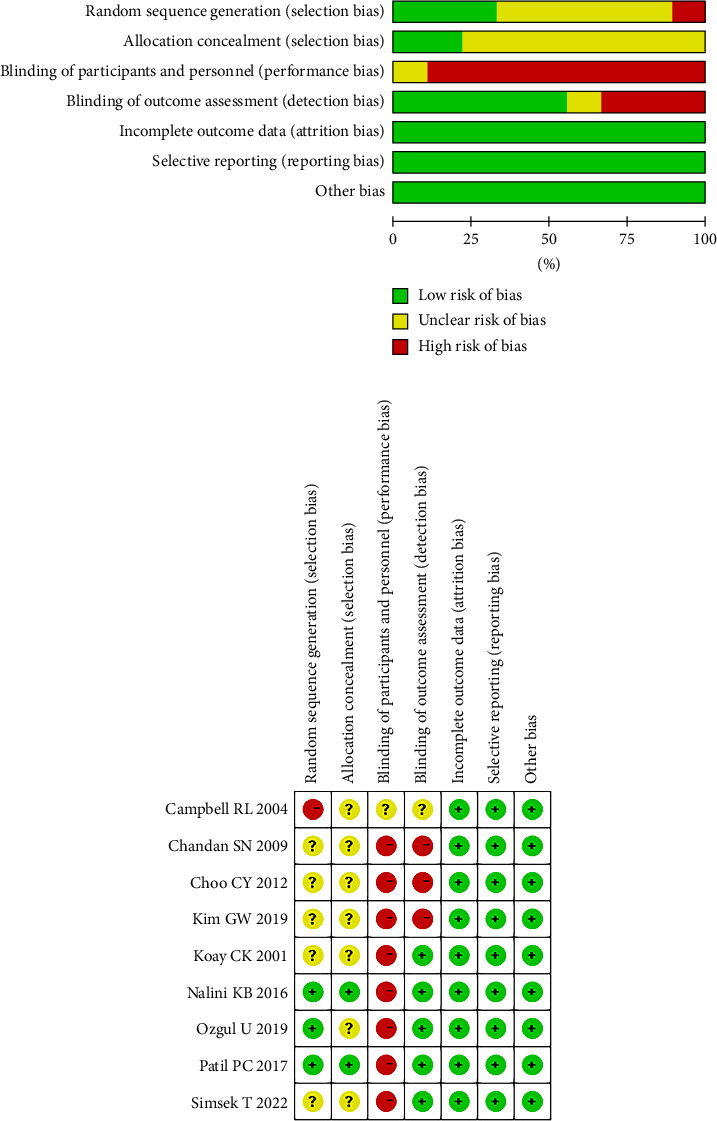
Summary of the risk of bias of the included trials.

The quality indicators of the studies included in this review are shown in Figure 2. Eight studies used random sequence generation. Nevertheless, the specific randomization methods used in five studies remain unstated [8, 10, 11, 13, 14]. Two studies implemented concealed allocation [12, 15]. Due to the inherent characteristics of the trial, the anesthesiologist tasked with inserting LMA and collecting intraoperative data was unable to maintain blinding. Three studies were identified as having a high risk of bias in this regard [8, 11, 14], and one study did not specify the associated risks [9]. Importantly, none of the studies reported incomplete outcome data or engaged in selective reporting. Eight studies received scores of 4 or higher on the modified Jadad scale, indicating a high level of quality. The relevant data are detailed in Table 3.

### 3.4. Fiberoptic Staging

**Figure 3:**
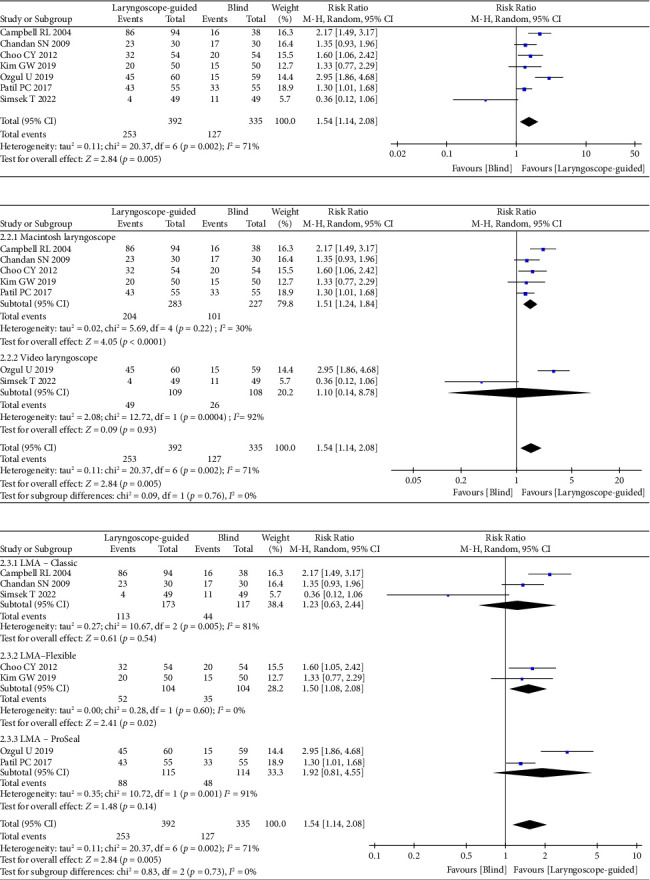
Forest plots of laryngoscope guidance versus blind on fiberoptic staging. (a) Forest plots of fiberoptic staging. (b) Subgroup analysis of fiberoptic staging by laryngoscope types. (c) Subgroup analysis of fiberoptic staging by LMA types.

A total of seven randomized trials that reported on fiberoptic staging were included in the analysis, comprising 392 patients in Group L and 335 patients in Group B [8–12, 14, 16]. The results indicated that Group L exhibited a higher fiberoptic staging classification of 4 than Group B (RR = 1.54; 95% CI: 1.14–2.08; *p* = 0.005). High heterogeneity was observed among the studies (*I*^2^ = 71%, *p* = 0.002) (Figure 3(a)). In a subgroup analysis examining the type of laryngoscope used during anesthesia, the results indicated that the incidence of fiberoptic staging classification of 4 in Group L was significantly higher than that in group B within the Macintosh laryngoscope subgroup (RR = 2.97; 95% CI 2.03 to 4.37; *p* < 0.001). A high heterogeneity was indicated (*I*^2^ = 70%, *p* = 0.009). Conversely, in the video-laryngoscope subgroup, there was no significant difference (RR = 1.10; 95% CI: 0.14–8.78; *p* = 0.93). A high heterogeneity was observed (*I*^2^ = 92%, *p* = 0.0004) (Figure 3(b)). A subgroup analysis of various types of laryngeal masks indicated that within the LMA–Flexible subgroup, Group L exhibited a higher incidence of Fiberoptic staging 4 compared to Group B (RR = 1.50; 95% CI: 1.08–2.08; *p* = 0.02; *I*^2^ = 0%, *p* = 0.60). In the LMA-Classic subgroup, the pooled outcomes indicated no significant difference between Group L and Group B (RR = 1.23; 95% CI: 0.63–2.44; *p* = 0.54; *I*^2^ = 81%, *p* = 0.005), as well as in the LMA–ProSeal subgroup (RR = 1.92; 95% CI: 0.81–4.55; *p* = 0.02; *I*^2^ = 0%, *p* = 0.60) (Figure 3(c)).

### 3.5. OLP, Success Rate at First Insertion, and Insertion Time

**Figure 4:**
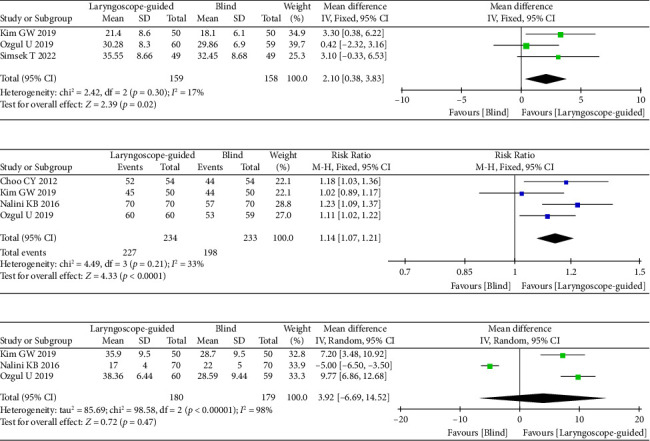
Forest plots of laryngoscope guidance versus blind on OLP, success rate at the first attempt, and insertion time. (a) Forest plots of OLP. (b) Forest plots of success rate at the first attempt. (c) Forest plots of insertion time.

Three studies [10, 11, 16] compared OLP, including 159 cases in Group L and 158 cases in Group B. The OLP of Group L was significantly higher than that of Group B (MD = 2.10 cmH_2_O; 95% CI: 0.38 cmH_2_O–3.83 cmH_2_O; *I*^2^ = 17%, *p*=0.02) (Figure 4(a)).

Four RCTs [11, 14–16] that reported on the success rate at the first insertion included 234 patients in Group L and 233 in Group B. The success rate for the first insertion in Group L was higher than that in Group B (RR = 1.14; 95% CI: 1.06–1.22; *I*^2^ = 33%, *p*=0.0005) (Figure 4(b)).

Three RCTs [11, 15, 16] that reported on insertion time included 180 patients in Group L and 179 in Group B. The insertion time of Group L was not significantly different from that of Group B (MD = 3.92 s; 95% CI: -6.69 s–14.52 s; *p*=0.47; *I*^2^ = 98%, *p* < 0.001) (Figure 4(c)). The muscle relaxant was used in the study [15]. After the study [15] was excluded from the analysis, the results changed; the insertion time in Group L was found to be significantly longer than that in Group B (MD = 8.76 s; 95% CI: 6.30 s–11.22 s; *p*=0.29; *I*^2^ = 12%, *p*=0.29).

### 3.6. Sore Throat and Hemorrhagic Staining

**Figure 5:**
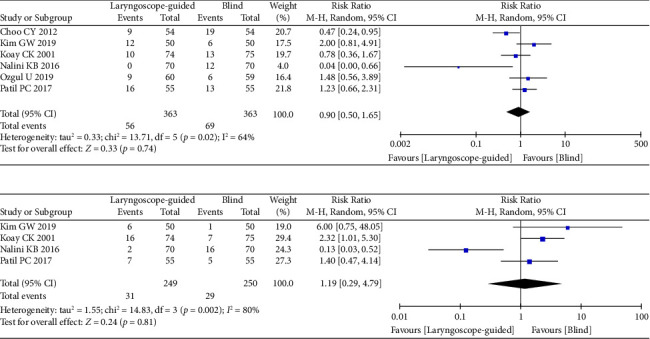
Forest plots of laryngoscope guidance versus blind on sore throat and blood staining. (a) Forest plots of sore throat. (b) Forest plots of blood staining.

A total of six RCTs [11–16] investigating sore throat included 56 patients in Group L and 69 patients in Group B. The incidence of sore throat in Group L was not significantly different from that in Group B (RR = 0.90; 95% CI: 0.50–1.65; *p*=0.74; *I*^2^ = 64%, *p*=0.02) (Figure 5(a)).

Four RCTs [11–13, 15] recorded blood staining, including 249 cases in Group L and 250 cases in Group B. The rate of blood staining in Group L was not significantly different from that in Group B (RR = 1.19; 95% CI: 0.29–4.79; *p*=0.81; *I*^2^ = 80%, *p*=0.002) (Figure 5(b)). The exclusion of the study [15] significantly reduced the heterogeneity associated with blood staining. Furthermore, the incidence of blood staining in Group L was higher than that in Group B (RR = 2.13; 95% CI: 1.14–4.00; *p*=0.02; *I*^2^ = 0%, *p*=0.45).

### 3.7. Investigation of Heterogeneity

High heterogeneity was observed in the comprehensive analysis of fiberoptic staging, insertion time, sore throat, and blood staining. The subgroup analysis of fiberoptic staging indicated that the type of laryngoscope and laryngeal mask contributed to the high levels of heterogeneity. Insertion time was assessed in three studies [11, 15, 16], with the use of muscle relaxants noted during LMA insertion in the study [15]. After the study [15] was excluded, the remaining two studies exhibited low heterogeneity. A total of six studies evaluated postoperative sore throat [11–16], with patients in articles [14, 15] undergoing digital techniques and dental surgery. After excluding two studies [14, 15], low heterogeneity was observed in the remaining four studies. Blood staining was reported in articles [11–13, 15], and low heterogeneity was observed in the remaining three studies after the exclusion of the study [15].

### 3.8. Bias in Academic Publications

The funnel plot of publication bias is shown in Appendix 2.

## 4. Discussion

The finding of this meta-analysis indicated that laryngoscope guidance resulted in a higher fiberoptic staging classification, significantly improved OLP, and increased the success rate on the first attempt. However, it did not demonstrate a reduction in insertion time compared to blind insertion among patients undergoing general anesthesia. Furthermore, no significant differences were observed in the incidence of sore throat or blood staining between the laryngoscope-guided and the blind insertion method.

The results of this meta-analysis indicated that the incidence of Fiberoptic staging classification 4 in Group L was significantly higher than that in Group B. This finding indicated that laryngoscope guidance technology facilitates the insertion of the laryngeal mask into the optimal anatomical position, ensuring adequate ventilation. However, the analysis also revealed a high degree of heterogeneity. Notably, removing individual studies did not significantly reduce the heterogeneity associated with fiberoptic staging. Given the substantial heterogeneity observed, a subgroup analysis was conducted to assess the influence of potential confounding variables. Using different laryngoscopes in clinical practice may introduce confounding factors; our analysis indicated that video laryngoscopes could contribute to these confounding variables. Specifically, the performance of the video laryngoscope did not show a significant difference compared to the blind insertion group. Furthermore, we performed a subgroup analysis considering the confounding factors associated with various types of LMAs used in clinical settings. The result revealed that the fiberoptic staging in Group L was higher than that in Group B for the subgroup using flexible laryngeal masks. Conversely, no significant differences in optical fiberoptic staging were observed between laryngoscope guidance and blind insertion methods within the LMA–Classic and LMA–ProSeal subgroups. Even though subgroup analyses were employed, high heterogeneity was still indicated. This was likely because different sizes of laryngeal masks were used in these trials. In addition, variations in induction methods, maintenance protocols, anesthetic depth, measurement standards, and patient sample sizes may have further contributed to the observed data heterogeneity.

Our meta-analysis indicated that the OLP of Group L was significantly higher than that of Group B. OLP is used to evaluate the appropriate placement of LMA. A higher value of OLP is generally indicative of a more stable ventilation. This study's results indicated that using a laryngoscope can lead to a higher OLP. This is advantageous for surgeries that require prolonged positive pressure ventilation. It is preferable to achieve successful insertion on the first attempt, as repeated attempts to insert LMA could increase the risk of airway trauma and may result in failure due to delays. Lee reported that using a laryngoscope can improve the placement of LMAs in adults [32]. However, the study found that laryngoscope-guided insertion did not increase the first-attempt success rate, which may be attributed to the limited sample size [11]. In this meta-analysis, the first-attempt success rate of Group L was higher than that of Group B. This might reduce the incidence of adverse complications caused by multiple insertion attempts. The insertion time was defined as when the device or laryngoscope blade was picked up until the first square wave of capnography was observed [16]. This meta-analysis revealed that the insertion time of Group L did not differ significantly from that of Group B; however, high heterogeneity was indicated. Further analysis showed that using muscle relaxants contributed to high heterogeneity and may influence LMA placement. Hattori et al. demonstrated that muscle relaxants may facilitate LMA insertion by relaxing the oropharyngeal muscle tissues [33]. Notably, following the exclusion of the study [15], the overall results were reversed, suggesting that the use of muscle relaxants may lead to an increased insertion time of the laryngeal mask when using a laryngoscope.

Tissue distortion, venous compression, and nerve injury may occur due to LMA insertion. Common complications associated with LMA insertion include sore throat and blood staining. Studies have indicated that laryngoscope-guided LMA insertion can reduce the rates of blood staining and sore throat [14, 15]. Conversely, other studies have indicated that laryngoscope-guided insertion did not significantly decrease the incidence of these complications [11–13]. Our meta-analysis showed that the incidence of sore throat and blood staining in Group L was comparable to that in Group B. However, a high heterogeneity was observed. Notably, the source of heterogeneity may be associated with the size of the LMA and the type of surgical procedure. Surgical procedures involving the oropharynx may influence the incidence of sore throat.

This study has several limitations. First, variations in laryngoscope types, LMA sizes and types, muscle relaxants use, and surgeries performed may have contributed to the observed data heterogeneity. Second, a critical factor influencing the success rate of the first insertion is the anesthesiologist's level of expertise in LMA insertion; however, this aspect was not addressed in any of the included trials. Third, excluding ongoing or unpublished research from comprehensive searches of published articles may introduce potential publication bias. Furthermore, while the three studies employed a randomization approach, some did not adequately describe this methodology in their results. Lastly, high-quality studies and further investigations are needed to validate our findings.

Laryngoscope guidance is an effective technique for facilitating the placement of LMA, which can be extensively used in clinical practice. The efficacy of LMA placement is influenced by the type of laryngoscope and the specific design of LMA employed. Therefore, selecting the most appropriate laryngoscope and laryngeal mask is crucial to enhance clinical outcomes and minimize the risk of complications.

## 5. Conclusion

In conclusion, our findings indicated that laryngoscope-guided insertion of LMA has several advantages, including ensuring correct placement of LMA as reflected in improved fiberoptic staging, improved OLP, and a higher insertion success rate. Furthermore, using laryngoscope guidance does not appear to increase the incidence of sore throat or blood staining in anesthetized patients. Consequently, our study advocates for using laryngoscope guidance in the insertion of LMA, as it demonstrates superior clinical outcomes compared to blind insertion.

## Figures and Tables

**Table 1:** The modified Jadad scale.

Classification	Description	Score
Randomization		
No	Semirandomized or quasirandomized trials	0
Unclear	Randomized trials without describing methods for generating random sequences	1
Yes	Random sequences produced by a computer or a random number table	2
Allocation concealment		
No	Regular grouping	0
Unclear	Only use of a random number table or other random assignment scheme	1
Yes	A method for assigning sequences without prediction	2
Blinding		
No	Use of double blindness without an appropriate method	0
Unclear	Only mentioned double blindness	1
Yes	A description of the specific and appropriate method of double blindness	2
Withdrawals or dropouts		
No	No description of withdrawal or dropouts	0
Yes	A description of withdrawal or dropouts	1

*Note:* Four criteria were used to assess the quality of the included studies: randomization (score: 0–2), blinding (score: 0–2), allocation concealment (score: 0–2), and withdrawals or dropouts (score: 0-1).

**Table 2:** Characteristics of the included studies.

First author	Year	Laryngeal mask type	Group	ASA	*N*	Type of operation	Outcomes
Choo	2012	LMA–Flexible	L	I-II	54	Dental surgery	①②③
			B	I-II	54		

Koay	2001	LMA–Classic	L	I-II	74	Elective orthopedic or general surgical procedures	③④
			B	I-II	75		

Simsek	2022	LMA–Classic	L	I-II	49	Ambulatory surgery or elective minor surgery	②⑤
			B	I-II	49		

Nalini	2016	LMA–ProSeal	L	I-II	70	Short surgical procedures with general anesthesia	①③④⑥⑦
			B	I-II	70		

Ozgul	2019	LMA–ProSeal	L	I-II	60	Elective surgery in the supine position under general anesthesia	①②③⑤⑥
			B	I-II	59		

Kim	2019	LMA–Flexible	L	I-II	50	Elective minor surgery or ambulatory surgery	①②③④⑤⑥
			B	I-II	50		

Chandan	2009	LMA–Classic	L	I-II	30	Oral and maxillofacial surgery, ENT and general surgery;	②
			B	I-II	30		

Campbell	2004	LMA–Classic	L	I-II	94	Office-based oral surgery; general surgery procedures of comparable complex	②
			B	I-II	38		

Patil	2017	LMA–ProSeal	L	I-II	55	Routine surgeries under general anesthesia	②③④⑦
			B	I-II	55		

*Note:* L, the laryngoscope-guided group; B, the blind insertion group; ①, success rate at first insertion; ②, fiberoptic assessment; ③, sore throat; ④, blood staining; ⑤, OLP (cmH_2_O); ⑥, insertion time; ⑦, the use of muscle relaxants.

Abbreviations: ASA, American Society of Anesthesiologists; LMA, laryngeal mask airway; N, number.

**Table 3:** Level of evidence and modified Jadad quality score.

References	Randomization	Allocation concealment	Blinding	Withdrawals and dropouts	Score
Choo, Koay, and Yoong [14]	1	0	1	2	4
Koay, Yoong, and Kok [13]	1	0	1	2	4
Simsek et al. [10]	1	0	1	2	4
Nalini et al. [15]	2	2	1	2	7
Ozgul et al. [16]	2	1	1	2	6
Kim et al. [11]	1	0	1	2	4
Chandan et al. [8]	1	0	1	2	4
Campbell et al. [9]	0	0	0	2	2
Patil et al. [12]	2	2	1	2	7

*Note:* Score: a scale of 4–7 indicated high quality, while a scale of 1–3 indicated inferior quality. Higher scores indicate higher quality on the scale, which ranges from 0 to 7.

## Data Availability

The data that support the findings of this study are available from the corresponding author upon reasonable request.

## Supporting Information

Supporting Information 1 Appendix 1: Search strategies.

Supporting Information 2 Appendix 2: Funnel plot for publication bias.

Supporting Information 3 Checklist: Reporting checklist for systematic review (with or without a meta-analysis).

## References

[1] Francksen H., Bein B., Cavus E. (2007). Comparison of LMA Unique, Ambu Laryngeal Mask and Soft Seal Laryngeal Mask during Routine Surgical Procedures. *European Journal of Anaesthesiology*.

[2] Seet E., Rajeev S., Firoz T. (2010). Safety and Efficacy of Laryngeal Mask Airway Supreme versus Laryngeal Mask Airway ProSeal: a Randomized Controlled Trial. *European Journal of Anaesthesiology*.

[3] De Montblanc J., Ruscio L., Mazoit J. X., Benhamou D. (2014). A Systematic Review and Meta‐analysis of the I‐gel^®^ vs Laryngeal Mask Airway in Adults. *Anaesthesia*.

[4] Maitra S., Baidya D. K., Bhattacharjee S., Khanna P. (2014). Evaluation of I-Gel(™) Airway in Children: a Meta-Analysis. *Pediatric Anesthesia*.

[5] Brain A. I. (1983). The Laryngeal Mask--a New Concept in Airway Management. *British Journal of Anaesthesia*.

[6] Van Zundert A. A. J., Gatt S. P., Kumar C. M., Van Zundert T., Pandit J. J. (2017). Failed Supraglottic Airway’: an Algorithm for Suboptimally Placed Supraglottic Airway Devices Based on Videolaryngoscopy. *British Journal of Anaesthesia*.

[7] Van Zundert A. A. J., Gatt S. P., Van Zundert T., Kumar C. M., Pandit J. J. (2022). Features of New Vision-Incorporated Third-Generation Video Laryngeal Mask Airways. *Journal of Clinical Monitoring and Computing*.

[8] Chandan S. N., Sharma S. M., Raveendra U. S., Rajendra Prasad B. (2009). Fiberoptic Assessment of Laryngeal Mask Airway Placement: a Comparison of Blind Insertion and Insertion with the Use of a Laryngoscope. *Journal of Maxillofacial and Oral Surgery*.

[9] Campbell R. L., Biddle C., Assudmi N., Campbell J. R., Hotchkiss M. (2004). Fiberoptic Assessment of Laryngeal Mask Airway Placement: Blind Insertion versus Direct Visual Epiglottoscopy. *Journal of Oral and Maxillofacial Surgery*.

[10] Simsek T., Saracoglu A., Sezen O., Cakmak G., Saracoglu K. T. (2022). Blind vs. Video-Laryngoscope-Guided Laryngeal Mask Insertion: A Prospective Randomized Comparison of Oropharyngeal Leak Pressure and Fiberoptic Grading. *Journal of Clinical Monitoring and Computing*.

[11] Kim G. W., Kim J. Y., Kim S. J., Moon Y. R., Park E. J., Park S. Y. (2019). Conditions for Laryngeal Mask Airway Placement in Terms of Oropharyngeal Leak Pressure: a Comparison between Blind Insertion and Laryngoscope-Guided Insertion. *BMC Anesthesiology*.

[12] Chikkapillappa M., Patil P., Pujara V. S., Anandswamy T. C., Parate L. H., Bevinaguddaiah Y. (2017). ProSeal Laryngeal Mask Airway Placement: A Comparison of Blind versus Direct Laryngoscopic Insertion Techniques. *Anesthesia: Essays and Researches*.

[13] Koay C. K., Yoong C. S., Kok P. (2001). A Randomized Trial Comparing Two Laryngeal Mask Airway Insertion Techniques. *Anaesthesia & Intensive Care*.

[14] Choo C. Y., Koay C. K., Yoong C. S. (2012). A Randomised Controlled Trial Comparing Two Insertion Techniques for the Laryngeal Mask Airway Flexible™ in Patients Undergoing Dental Surgery. *Anaesthesia*.

[15] Nalini K. B., Shivakumar S., Archana S., Sandhya Rani D. C., Mohan C. R. (2016). Comparison of Three Insertion Techniques of ProSeal Laryngeal Mask Airway: A Randomized Clinical Trial. *Journal of Anaesthesiology Clinical Pharmacology*.

[16] Ozgul U., Erdil F. A., Erdogan M. A. (2019). Comparison of Videolaryngoscope-Guided versus Standard Digital Insertion Techniques of the ProSeal™ Laryngeal Mask Airway: a Prospective Randomized Study. *BMC Anesthesiology*.

[17] Maynard S. L., Kao R., Craig D. G. (2016). Impact of Personal Protective Equipment on Clinical Output and Perceived Exertion. *Journal of the Royal Army Medical Corps*.

[18] Keller C., Brimacombe J., Pühringer F. (2000). A Fibreoptic Scoring System to Assess the Position of Laryngeal Mask Airway Devices. Interobserver Variability and a Comparison between the Standard, Flexible and Intubating Laryngeal Mask Airways. *Ains Anästhesiologie Intensivmedizin Notfallmedizin Schmerztherapie*.

[19] Brimacombe J., Berry A. (1993). A Proposed Fiber-Optic Scoring System to Standardize the Assessment of Laryngeal Mask Airway Position. *Anesthesia & Analgesia*.

[20] Kumar C. M., Van Zundert T. C., Seet E., Van Zundert A. A. (2021). Time to Consider Supraglottic Airway Device Oropharyngeal Leak Pressure Measurement More Objectively. *Acta Anaesthesiologica Scandinavica*.

[21] Van Zundert A. A. J., Kumar C. M., Van Zundert T., Gatt S. P., Pandit J. J. (2021). The Case for a 3rd Generation Supraglottic Airway Device Facilitating Direct Vision Placement. *Journal of Clinical Monitoring and Computing*.

[22] Keller C., Brimacombe J. R., Keller K., Morris R. (1999). Comparison of Four Methods for Assessing Airway Sealing Pressure with the Laryngeal Mask Airway in Adult Patients. *British Journal of Anaesthesia*.

[23] Kjaergard L. L., Villumsen J., Gluud C. (2001). Reported Methodologic Quality and Discrepancies between Large and Small Randomized Trials in Meta-Analyses. *Annals of Internal Medicine*.

[24] Brimacombe J., Berry A., Brain A. I. (1996). Optimal Intracuff Pressures with the Laryngeal Mask. *British Journal of Anaesthesia*.

[25] Kim D. H., Park J. Y., Yu J. (2020). Positive End-Expiratory Pressure Increases Arterial Oxygenation in Elderly Patients Undergoing Urological Surgery Using Laryngeal Mask Airway in Lithotomy Position. *Journal of Clinical Monitoring and Computing*.

[26] Beleña J. M., Ochoa E. J., Núñez M., Gilsanz C., Vidal A. (2015). Role of Laryngeal Mask Airway in Laparoscopic Cholecystectomy. *World Journal of Gastrointestinal Surgery*.

[27] Lai C. J., Liu C. M., Wu C. Y., Tsai F. F., Tseng P. H., Fan S. Z. (2017). I-gel Is a Suitable Alternative to Endotracheal Tubes in the Laparoscopic Pneumoperitoneum and Trendelenburg Position. *BMC Anesthesiology*.

[28] Weber U., Oguz R., Potura L. A., Kimberger O., Kober A., Tschernko E. (2011). Comparison of the I-Gel and the LMA-Unique Laryngeal Mask Airway in Patients with Mild to Moderate Obesity during Elective Short-Term Surgery. *Anaesthesia*.

[29] Raman R., Prabha R., Khan M. P., Kaushal D., Siddiqui A. K., Abbas H. (2018). Comparison of I-Gel for General Anesthesia in Obese and Nonobese Patients. *Saudi Journal of Anaesthesia*.

[30] Damayanthi E. D., Kholinne E., Singjie L. C., Sakti M., Anesstesia I. J. (2024). Combined Anterior Cruciate Ligament Reconstruction (ACLR) and Lateral Extra-articular Tenodesis through the Modified Lemaire Technique versus Isolated ACLR: A Meta-Analysis of Clinical Outcomes. *Rev Bras Ortop (Sao Paulo)*.

[31] Moher D., Shamseer L., Clarke M. (2015). Preferred Reporting Items for Systematic Review and Meta-Analysis Protocols (PRISMA-P) 2015 Statement. *Systematic Reviews*.

[32] Lee J. J. (1989). Laryngeal Mask and Trauma to Uvula. *Anaesthesia*.

[33] Hattori K., Komasawa N., Miyazaki Y., Kido H., Deguchi S., Minami T. (2016). Muscle Relaxant Facilitates I-Gel Insertion by Novice Doctors: A Prospective Randomized Controlled Trial. *Journal of Clinical Anesthesia*.

