# Supplementary material for: Comparison of Laryngoscope-Guided Insertion and Standard Blind Insertion of the Laryngeal Mask Airway: A Systematic Review and Meta-Analysis
Source: Anesthesiol Res Pract. 2025 Mar 18;2025:1224567. doi: 10.1155/anrp/1224567 (PMC11936533; doi:10.1155/anrp/1224567)
Supplement: Supporting Information 1 — Appendix 1: Search strategies. [file 1224567.f1.docx]

**Appendix1 Search strategies**

**Search strategy of PubMed as follows:**

#1 "Laryngeal Masks"[Mesh] OR laryngeal mask airway*[Title/Abstract] OR laryngeal

mask*[Title/Abstract] OR aryngeal mask*[Title/Abstract] OR arynx

mask*[Title/Abstract] OR LMA[Title/Abstract]

#2 Laryngoscope[Title/Abstract]

#3 insertion[Title/Abstract] OR placement[Title/Abstract]

#4 #1and #2 and #3

#5 random*[Title/Abstract] OR blind*[Title/Abstract] OR singleblind*

[Title/Abstract] OR doubleblind* [Title/Abstract] OR trebleblind*[Title/Abstract]

OR tripleblind*[Title/Abstract]

#6 "Clinical Trials as Topic"[Mesh]

#7 #5OR #6

#8 #4 and #7

**Search strategy of Cochrane library as follows:**

#1 MeSH descriptor: [laryngeal masks] explode all trees

#2 ("laryngeal mask airways"):ti,ab,kw OR ("laryngeal masks"):ti,ab,kw OR

("aryngeal masks"):ti,ab,kw OR ("arynx masks"):ti,ab,kw OR ("laryngeal mask

airway"):ti,ab,kw OR ("laryngeal mask"):ti,ab,kw OR ("aryngeal mask"):ti,ab,kw OR ("arynx mask"):ti,ab,kw OR (LMA):ti,ab,kw

#3 (Laryngoscope):ti,ab,kw

#4 (insertion):ti,ab,kw OR (placement):ti,ab,kw

#5 #1 and #2 and #3 and #4

**Search strategy of Web of Science as follows:**

#1 TS=("laryngeal mask airways" OR "laryngeal masks" OR "aryngeal masks" OR

"arynx masks" OR "laryngeal mask airway" OR "laryngeal mask" OR "aryngeal mask"

OR "arynx mask" OR LMA)

#2 TS= (Laryngoscope)

#3 TS=( insertion OR placement)

#4 #1 and #2 and #3

#5 TS=(random* OR blind* OR singleblind* OR doubleblind* OR trebleblind* OR

tripleblind*)

#6 #4 AND #5

**Search strategy for Ovid Medline as follows:**

Ovid MEDLINE(R) and Epub Ahead of Print, In-Process, In-Data-Review & Other Non-Indexed Citations and Daily <1946 to September 01, 2023>

1 Laryngeal Masks/ 5598

2 tube*.mp. 578546

3 mask*.mp. 110067

4 2 or 3 684850

5 laryn*.mp. 124896

6 (laryn* adj2 (tube* or mask*)).mp. 8585

7 LMA.mp. 3771

8 oropharyn*.mp. 32566

9 cuff*.mp. 39917

10 (cuff* adj3 oropharyn*).mp. 117

11 epiglottic.mp. 1166

12 supraglottic.mp. 5048

13 11 or 12 6074

14 airway*.mp. 209647

15 ((epiglottic or supraglottic) adj3 airway*).mp. 1449

16 i-gel.mp. 818

17 i gel.mp. 818

18 16 or 17 818

19 streamlined liner of the pharynx airway.mp. 23

20 SLIPA.mp. 38

21 19 or 20 41

22 1 or 6 or 7 or 10 or 15 or 18 or 21 11388

23 exp Laryngoscope/ 4136

24 insertion.mp. 174263

25 placement.mp. 156138

26 24 or 25 317468

27 22 and 23 and 26 45

28 randomized controlled trial.mp. or Randomized Controlled Trial/ 644544

29 controlled clinical trial.mp. or Controlled Clinical Trial/ 113999

30 (randomized controlled trial or Randomized Controlled Trial or (controlled clinical trial or Controlled Clinical Trial)).pt. 689618

31 randomized.ab. 615476

32 placebo.ab. 241091

33 randomly.ab. 415637

34 trial.ab. 662177

35 30 or 31 or 32 or 33 or 34 1614812

36 35 not (animals not (humans and animals)).sh. 1472047

37 27 and 36 16
