# Supplementary material for: Comparison of Laryngoscope-Guided Insertion and Standard Blind Insertion of the Laryngeal Mask Airway: A Systematic Review and Meta-Analysis
Source: Anesthesiol Res Pract. 2025 Mar 18;2025:1224567. doi: 10.1155/anrp/1224567 (PMC11936533; doi:10.1155/anrp/1224567)
Supplement: Supporting Information 2 — Appendix 2: Funnel plot for publication bias. [file 1224567.f2.pdf]

Appendix2 Funnel plot for publication bias

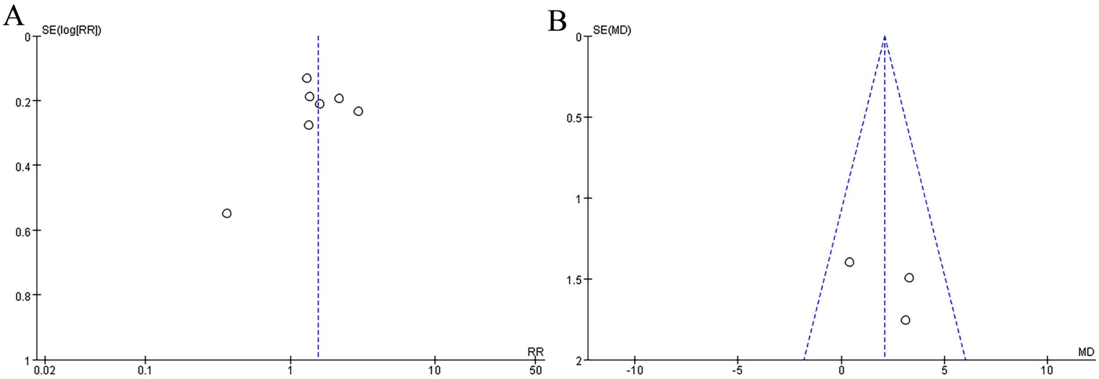

Funnel plot for publication bias of the included studies. (A) Distribution of studies included in fiberoptic staging. (B) Distribution of studies included in OLP.
